# How to approach supervisors for research opportunities

**DOI:** 10.1016/j.amsu.2016.01.022

**Published:** 2016-01-22

**Authors:** Daniyal J. Jafree, Katharine Whitehurst, Shivanchan Rajmohan

**Affiliations:** a University College London Medical School, UK; b Imperial College London, UK

**Keywords:** Research, Undergraduate, Graduate, Supervisor

## Abstract

In this article, we use our experiences to provide tips for contacting potential supervisors, what to expect from them and how to approach them for research opportunities. With appropriate planning, you will be surprised by the number of prestigious academics who would be willing for you to join their research group, and to get you involved in a research project.

## Highlights

• Contacting potential supervisors is a time consuming process that requires a great deal of organisation.
• Be proactive in your approach.
• The first email is very important, as is the meeting that may follow.
• Choose carefully and take a holistic approach when choosing your supervisor to ensure you have the best possible research experience.

## 1 Why contact a supervisor and how can I do it?

Undergraduate and postgraduate students may be very keen on research, and eager to take up opportunities benefitting them in the future on their paths to become academic clinicians, surgeons and beyond.

After selecting a particular topic of interest to pursue research in, the selection of a good supervisor is critical, as the working relationship you build with your supervisor can determine the success of this research.

To acquire the email addresses of some of the country's top lecturers and academics, all that is required is to go onto their respective university websites. It is customary to email these potential supervisors for research opportunities: be it for summer research, a future BSc project or a prospective PhD.

## 2 Planning

There are several considerations one should make before contacting potential supervisors. It is important to carefully plan both the people you contact, and the emails you send:

• Firstly, identify your own interests. What are you aiming to achieve with your research e.g.: Are you looking to contact supervisors because you would like to undertake your BSc Project on deep brain stimulation? Or perhaps a PhD in neural tube defects?
• Identify academics within your institution relevant to your chosen interest. Your university may have a research portal that you can use to find researchers within particular fields. An example is University College London's (UCL) IRIS Research Portal, which makes it really easy to draw up a list of potential supervisors for research you are interested in getting involved with.
• Draw up a list of potential supervisors and perform relevant background reading about each one. Sometimes researchers can have their own websites in which you can find more details about their work and research interests. It may also be wise to read some of their latest publications, accessible via PubMed. Not only does this give you a better idea as to their speciality of work, but it also shows commitment if you do establish contact with them.

## 3 Is it okay to contact other potential supervisors?

Medical students, particularly at an early career stage, may have multiple interests, and may wish to enquire about potential research opportunities with different supervisors. However, when emailing and meeting with supervisors, it should be made clear from the outset that other supervisors are also being contacted. This transparency is important to avoid any future misunderstanding. If a careful approach is taken, most supervisors are understanding. A good supervisor will appreciate that students are simply expressing interest, and enquiring with different supervisors to determine if potential projects and research experience align more strongly with their interests and aspirations.

## 4 How to approach the first email

Think of the first email to the supervisor as a cover letter. The email should demonstrate why you are a suitable student for the chosen research, and why the supervisor should take you on as a research student. The following points should be considered prior to composing the email. These points may seem trivial, but could make the difference between obtaining a reply or not:

• Email etiquette is imperative. Start the email with “Dear” and end it with “Kind Regards”. Spelling and grammar errors are to be avoided.
• Good formatting is also essential. Choose an appropriate font size and style. Allow for adequate spacing between lines and between paragraphs.
• A clear subject title should be considered. This should attract a supervisor's attention to you as a potential research student. This may relate to the intended research position i.e.: ‘Interest in Wellcome Trust PhD in Regenerative Medicine’

Moreover, the email should be structured coherently. Through the following points, we propose a structure which can be implemented when contacting potential supervisors for research opportunities.

• Introduction
  ○ State who you are and what you do, or what you intend to do. For example “My name is Dan, I am a 2^nd^ year medical student looking to undertake a BSc in Surgical Sciences next academic year”
  ○ It might be beneficial to state how you came to hear about the supervisor i.e.: did you attend one of his or her lectures? Mention whether you found it particularly interesting! Additionally, if the supervisor was recommended by another contact, then consider mentioning this here.
  ○ It is useful to put forward an objective e.g.: if you are thinking of applying for a BSc Prize, Scholarship or aspire to present your work at a particular conference, then it is worth mentioning this.
• Body
  ○ The main part of the email is arguably the most important. Here, as with any cover letter, you should promote yourself as a strong candidate for research in the supervisor's lab.
  ○ It is imperative to attach a curriculum vitae (CV) with the email. Highlight the most important points of your CV, such as research experience and commitment to research. Previous presentations and publications are useful to demonstrate a strong track record, and show yourself to be a good potential candidate for their research group.
  ○ It is useful to mention your interest in the supervisor's field, and why you want to undertake research in their lab. You may wish to mention any publications of theirs you have read.
  ○ If the research you intend to pursue requires funding, state the source (e.g. self-funded, received a grant, etc.). However this may not always be applicable or relevant.
• Conclusion
  ○ The end of the email should reinforce and summarise why you think you are suitable for the research role. You may even wish to request a meeting with the supervisor to discuss the projects he or she will have available for you.
  ○ It would be worth mentioning support you may have received i.e.: if your department has nominated you for an award, it would greatly strengthen your claim.
  ○ Ideas and aspirations for the future are useful to add here i.e.: I hope to develop into an academic neurosurgeon with a focus on deep brain stimulation.

## 5 Why haven't I got a reply?

Response rates from supervisors are variable, as many students underestimate the importance of the first email in impressing themselves as viable candidates for research. Many supervisors are extremely busy and have other commitments outside their academia. Therefore, it is okay to resend the email after a period of time, one week for example, as it is easy for your email to be ‘buried’ beneath more recent emails received by the recipient.

## 6 The meeting

It is not uncommon for potential supervisors to offer a meeting with themselves or their colleagues to further discuss your interests, aspirations for your research and the possibility of performing research under their supervision. If a supervisors is particularly busy, he or she may request that you organize a meeting with a different member of staff within their research group instead. This is both an opportunity to learn more about the department and its research, and to learn more about the potential supervisor and whether they are the right choice for you!

• Before the Meeting
  ○ Though it is not always necessary, it is useful to read some of the publications that your supervisor has contributed to. It sounds impressive in a meeting if you already have knowledge in the subject field, and it leaves more time in the meeting to discuss topics of your particular interest rather than having to explain previous work to you to put the research in context
  ○ Reread your email. Your potential supervisor may ask you some questions regarding what you have mentioned i.e.: how did you develop your interest of deep brain stimulation?
  ○ Sometimes, it can be appropriate to bring examples of your previous work For example, it may be advantageous for students to bring printouts of their previous publications to their meetings.
  ○ Do not underestimate the importance of a good first impression. Appear well-dressed, enthusiastic and motivated!
• During the Meeting
  ○ The meeting is a great way to assess your potential supervisor and whether they are the right choice for you. Assess how engaging the meeting is, whether the topic is really what you thought it would be and whether there is scope for a project you want to undertake with this supervisor.
  ○ You should also be considering other aspects of the supervisor. Are they listening to you? How much are they interested in you as a prospective student? Though these seem like minor points, but you should be considering these, particularly if the research is part of a BSc, MSc or PhD. In these cases, you will spend a substantial amount of time under their supervision.
  ○ How much time is the supervisor willing to put into your development? Many high-proline supervisors will likely be extremely busy, and will not be able to contribute a significant amount of time to your development as a researcher. We strongly believe the best supervisors to be reputable in their field, but still able to dedicate time to teach you the appropriate research techniques, arranging meetings with you to monitor progress, teaching you how to write dissertations and so on.
  ○ Consider further opportunities that your supervisor may offer you – will your supervisor allow you to contribute to some of their manuscripts for publication? Will he or she allow you to undertake further research opportunities and become involved in other projects within their research group?
  ○ For laboratory research, it may be appropriate to ask the supervisor if you can look around the laboratory and meet the research team, as it is likely that a majority of your time will be spent in the laboratory, and majority of the ‘hands-on’ supervision will be by other members of the laboratory team.
• After the Meeting
  ○ Consider writing all the information you gathered from the meeting down onto a table, such that when after meeting several supervisors with several available projects you can compare and contrast each to select one for your research intentions.
  ○ If possible, it is worth using your contacts or other sources to find supervisors. If several previous students particularly recommend a particular supervisor, then it is a very good sign!

## 7 Selecting your supervisor

The selection of a supervisor is a personal decision This decision should be considered carefully, taking the aforementioned points into account and weighing them up. The decision is even more critical if you are electing to spend a longer amount of time for the research, such as for an MSc or PhD. Choose carefully!

## Conflicts of interest

None to declare.

## Funding

The authors received no funding for this article.

## Ethical approval

No ethical approval was necessary.

## Author contribution

DJ conceived the article and created the first draft. KW and SR were responsible for critical review and approval of the final draft.

## Guarantor

DJ – the corresponding author.

